# Evaluation of the therapeutic effect of adaptive deep brain stimulation on motor symptoms and sleep disturbances in Parkinson’s disease and construction of a response prediction model

**DOI:** 10.3389/fneur.2025.1580273

**Published:** 2025-10-07

**Authors:** Mingming Su, Lihong Qu, Xin Wang, Bao Wang, Nan Li, Xuelian Wang, Zhaohui Zheng

**Affiliations:** ^1^Department of Neurosurgery, Tangdu Hospital, Air Force Medical University, Xi’an, Shaanxi, China; ^2^Department of Anesthesiology, Tangdu Hospital, Air Force Medical University, Xi’an, Shaanxi, China

**Keywords:** Parkinson’s disease, adaptive deep brain stimulation, motor symptoms, sleep disorders, generalized linear mixed model

## Abstract

**Background:**

Parkinson’s disease patients often experience symptoms such as motor impairments and sleep disturbances. This study aims to evaluate the efficacy of adaptive deep brain stimulation therapy in improving motor symptoms and sleep disorders in patients with Parkinson’s disease.

**Methods:**

This retrospective cohort study included 280 patients with Parkinson’s disease. Baseline data were analyzed to assess changes in motor symptoms and sleep disorders before and after treatment. Factors influencing treatment efficacy were explored using univariate and multivariate logistic regression analyses, based on which a response prediction model was constructed. A generalized linear mixed model was then employed to examine interactions between the response model and other variables.

**Results:**

After treatment, the Unified Parkinson’s Disease Rating Scale Part II (UPDRS II), Unified Parkinson’s Disease Rating Scale Part III (UPDRS III), Parkinson’s Disease Sleep Scale (PDSS), Pittsburgh Sleep Quality Index (PSQI), and Epworth Sleepiness Scale (ESS) scores in the observation group (adaptive deep brain stimulation, aDBS) were significantly lower than those in the control group, indicating better motor and non-motor symptom control. In contrast, the Mini-Mental State Examination (MMSE) score was significantly higher in the observation group, suggesting improved cognitive function. Age, body mass index (BMI), disease duration, Hoehn and Yahr stage, smoking history, and baseline neutrophil-to-lymphocyte ratio (NLR) were negatively associated with symptom improvement. In contrast, adaptive deep brain stimulation (aDBS) treatment showed a significant positive association with symptom improvement. The predictive model constructed based on blood biomarkers, demographic factors, and treatment response demonstrated good predictive performance for clinical improvement. Furthermore, generalized linear mixed model (GLMM) analysis revealed that the response model exerted an antagonistic effect on BMI and high-density lipoprotein (HDL) levels, and a synergistic effect on the platelet-to-lymphocyte ratio (PLR).

**Conclusion:**

The effectiveness of adaptive deep brain stimulation (aDBS) therapy in improving motor symptoms, sleep disorders, and quality of life in patients with Parkinson’s disease is superior to that of conventional treatment. Factors such as patient age, body mass index (BMI), disease duration, Hoehn-Yahr stage, and baseline blood marker levels can influence the efficacy of aDBS. The constructed response model effectively predicts symptom improvement and offers valuable guidance for clinical treatment decisions.

## 1 Introduction

Parkinson’s disease (PD) is a common neurodegenerative disorder characterized by motor symptoms such as tremors, rigidity, and bradykinesia (1). These symptoms significantly impair patients’ daily functioning and quality of life (2, 3). However, PD is not limited to motor manifestations; an increasing number of studies highlight sleep disorders as prevalent and severe non-motor symptoms in patients with Parkinson’s disease (4, 5). These sleep disturbances include insomnia, early awakening, nocturnal awakenings, and rapid eye movement sleep behavior disorder (RBD), which pose substantial challenges to patients’ physical and mental health. Moreover, they may exacerbate cognitive decline and psychological issues, further diminishing quality of life.

Traditional treatment methods primarily rely on pharmacotherapy; however, many patients gradually develop drug resistance or experience side effects after long-term medication, resulting in poor symptom control (6). Deep brain stimulation (DBS) has emerged as an effective treatment and has been widely applied in patients with drug-resistant Parkinson’s disease in recent years (7). DBS is a surgical intervention that involves implanting electrodes in specific brain regions to deliver electrical stimulation, thereby modulating neural activity and alleviating symptoms (8). Adaptive deep brain stimulation (aDBS), an innovative advancement of DBS, differs by adjusting stimulation parameters in real-time according to the patient’s motor symptoms, leading to improved therapeutic outcomes and reduced side effects (9, 10).

Previous studies have demonstrated that adaptive deep brain stimulation (aDBS) can effectively improve motor symptoms in Parkinson’s disease patients (11), although most research has focused primarily on optimizing aDBS parameter settings (12, 13). Furthermore, the improvement of motor symptoms and sleep disorders in Parkinson’s disease is influenced not only by treatment methods but also by other factors such as patient age, disease duration, and disease severity. Previous studies have not considered these factors that can affect the efficacy of aDBS. This study aims to evaluate the effectiveness of adaptive deep brain stimulation (aDBS) in improving motor symptoms and sleep disturbances in patients with Parkinson’s disease. We performed univariate and multivariate logistic regression analyses to identify significant demographic and blood biomarker factors influencing the efficacy of aDBS. Based on these significant factors, we constructed a response prediction model and further analyzed the interaction between the response model and other variables using a generalized linear mixed model, with the goal of providing a more scientific basis for individualized treatment.

## 2 Materials and methods

### 2.1 Patients

This retrospective study included 280 patients with Parkinson’s disease (PD) who visited our hospital between January 2019 and June 2022 as the research subjects, and divided them into a control group (*n* = 140) and an observation group (*n* = 140) according to the random number table method. The control group received conventional drug treatment, while the observation group received aDBS treatment in addition to conventional drug treatment. Inclusion criteria: After examination of blood cerebrospinal fluid, positron emission computed tomography (PET), single-photon emission computed tomography (SPECT), all the patients were diagnosed with PD; patients with normal heart, liver, kidney and other functions; The patient or his/her family member signs an informed consent form. Exclusion criteria: patients with allergic constitution or allergy to drugs in this study; patients with contraindications for DBS treatment or who could not tolerate the treatment; Patients with peripheral nervous system diseases, or with diabetes complicated by clinically diagnosed peripheral neuropathy, or other diseases affecting peripheral sensory function; Patients with severe psychiatric disorders (e.g., schizophrenia, major depressive disorder) or significant psychological disturbances (e.g., anxiety disorders, cognitive impairment) that result in poor treatment compliance; Patients with hyperthyroidism and essential tremor; patients with malignant tumor; Patients with secondary parkinsonism caused by drugs, viral encephalitis, brain trauma, carbon monoxide poisoning, etc.

### 2.2 Treatment methods

The control group was treated with conventional drugs: the patients were treated with Levodopa and Benserazide (Shandong Xinhua Pharmaceutical Co., Ltd., GYZZ H10930198) at an initial dose of 62.5 mg/time, twice a day, and then the dose was gradually increased to 250 mg/time, three times a day, in addition to the combined use of piribedil sustained-release tablets (Servier (Tianjin) Pharmaceutical Co., Ltd., GYZZ J20090075), amantadine (Jiangsu Pengqiao Pharmaceutical Co., Ltd., GYZZ H32023575), trihexyphenidyl hydrochloride (Changzhou Kangpu Pharmaceutical Co., Ltd., GYZZ H32022135) and other anti-PD drugs.

The observation group receives adaptive deep brain stimulation (aDBS) treatment in addition to the standard treatment: 12 h before aDBS treatment, medications are discontinued, and the Leksell stereotactic frame (Elekta, Sweden) was installed until local anesthesia acted, attention was paid to overlap the midsagittal plane with the midsagittal plane of the head during installation, and the baseline of the frame and the projection line of the anterior–posterior (AC-PC) line body surface maintained a parallel relationship. After the installation of the base frame, computed tomography (CT) thin-section scanning localization was performed, while the surgical planning system was imported into it to establish the coordinate system. Preoperative magnetic resonance imaging (MRI) images and CT images were fused with the help of SurgiPlan planning system (Elekta, Sweden) to fuse the subthalamic nucleus target, while the puncture path was designed, taking care to avoid sulci and intraventricular vessels. The puncture points on the scalp were marked according to the surgical plan, and after local anesthesia, a 1 mm microdrill bit was used to pass through the scalp to find the exact entry point into the skull on the skull, and after scalp incision, skull drilling was performed to incise the dura mater. The arcuate arch of the stereotactic system was installed, the system was advanced, and the microelectrodes were slowly pushed in. LeadPoint (Medtronic, United States) was used to record the neuronal cell discharges at different positions to determine the location of the subthalamic nucleus. If the recorded subthalamic nucleus signal was not satisfactory, the micromotor was slowly withdrawn and the coordinates were readjusted. At this point, the micromotor is implanted into the subthalamic nucleus and connected to the aDBS system. The system continuously monitors brain activity and automatically adjusts stimulation parameters—frequency, intensity, and pulse width—based on real-time neural signal changes. Through a closed-loop feedback mechanism, the stimulator adjusts its intensity and frequency according to the brain activity and the patient’s symptom feedback. Once the electrode position is confirmed, and the aDBS system is successfully installed, the physician adjusts the stimulation strength in real-time to determine the optimal treatment plan. After ensuring the electrode is in the best position and the patient’s symptoms have improved satisfactorily, the stimulator is implanted under general anesthesia beneath the left clavicle, with the lead wire extended through a subcutaneous tunnel in the neck.

Both groups were continuously treated for 6 months.

### 2.3 Data collection

Baseline data of patients were collected, including age, gender (male, female), body mass index (BMI), course of disease, smoking history (mild, moderate, severe), drinking history (mild, moderate, severe), complications including cardiovascular disease (yes, no), diabetes (yes, no), digestive system disease (yes, no), urinary system disease (yes, no), musculoskeletal disease (yes, no), and mental disease (yes, no), Hoehn-Yahr classification (phase I, II, and III), baseline C-reactive protein (CRP, unit: mg/L), White blood cell count (WBC, unit: 10^9/L), neutrophil to lymphocyte ratio (NLR), platelet to lymphocyte ratio (PLR), monocyte to lymphocyte ratio (MLR), triglycerides (TGL, unit: mmol/L), Low density lipoprotein cholesterol (LDL, unit: mmol/L) and high-density lipoprotein cholesterol (HDL, unit: mmol/L). Before and after treatment, the Unified Parkinson’s Disease Rating Scale Part II (UPDRS II) and Part III (UPDRS III) scores, as well as the Mini-Mental State Examination (MMSE) scores, PDSS (Parkinson’s Disease Sleep Scale, total score 0–120, with higher scores indicating more severe sleep problems), PSQI (Pittsburgh Sleep Quality Index, total score 0–21, with higher scores indicating poorer sleep quality), and ESS (Epworth Sleepiness Scale, total score 0–24, with higher scores indicating greater daytime sleepiness) scores were used to assess the motor function and sleep disorders of both groups of patients. We define a decrease of more than 10% in UPDRS II score or more than 20% in UPDRS III score as significant improvement in motor function, a decrease of more than 10 points in PDSS score, a decrease of more than 3 points in PSQI score, and a decrease of more than 3 points in ESS score as significant improvement in sleep disorders.

### 2.4 Statistical analysis

This study used R4.4.0 software for data analysis. All measurement data are expressed as median (minimum-maximum), and *t*-test is used for inter group comparison. For non-normally distributed metric data, Mann-Whitney U test is used. Categorical variables are expressed in frequency (percentage), and chi square tests are used for inter group comparisons. We will divide the collected baseline data into two categories: demographic and blood biomarkers, and conduct univariate and multivariate logistic regression analyses with treatment methods. The dependent variables are set to show significant improvement in both motor function and sleep disorders. Analyze and calculate the sensitivity, specificity, and area under the curve (AUC) of the model using receiver operating characteristic (ROC) curves, and evaluate the accuracy and effectiveness of the model in predicting improvements in motor symptoms and sleep disorders. The generalized linear mixed model (GLMM) was used to analyze the interaction between the response model and other influencing factors (such as BMI, HDL, etc.), and random effects were also added, that is, the individual factors of the patient were treated as random effects.

## 3 Results

### 3.1 Demographic and baseline blood marker characteristics of Parkinson’s disease patients

In this study, a total of 280 Parkinson’s disease patients were included, with a median age of 62 years and an age range of 46 to 81 years. Male patients account for 62.5% and female patients account for 37.5%. The median BMI of the patient is 30.0, ranging from 24.4 to 35.6. The median duration of the patient’s illness is 6.4 years, ranging from 1.0 to 11.8 years. In terms of smoking, 72.5% of patients are light smokers, 21.07% are moderate smokers, and 6.43% are heavy smokers; In terms of alcohol consumption, 83.57% of patients are light drinkers, 12.86% are moderate drinkers, and 3.57% are heavy drinkers. Patients with cardiovascular diseases accounted for 38.93%, patients with diabetes 26.43%, patients with digestive system diseases 42.86%, patients with urinary system diseases 33.93%, patients with musculoskeletal diseases 26.43%, and patients with mental diseases 14.29%. In Hoehn Yahr staging, 40% of patients are in stage I, 41.43% are in stage II, and 18.57% are in stage III. This indicates that the majority of patients are in the early stage (Table 1). At baseline, blood markers showed that most blood indicators such as C-reactive protein (CRP), white blood cells (WBC), neutrophil to lymphocyte ratio (NLR), etc. did not show significant differences between the two groups, while triglycerides (TGL) were close to significant levels (*p* value of 0.0753) (Table 2).

**Table 1:** Demographic characteristics of patients with Parkinson’s disease in the control and observation groups.

Variables	All patients (*n* = 280)	Control group (*n* = 140)	Observation group (*n* = 140)	*P*-value
Age (year)	62 (46–81)	62 (46–81)	62 (46–80)	0.852
Gender				0.3233773
Male	175 (62.5%)	92 (65.71%)	83 (59.29%)	
Female	105 (37.5%)	48 (34.29%)	57 (40.71%)	
BMI	30.0 (24.4–35.6)	29.6 (24.4–35.6)	30.4 (24.7–35.5)	0.874
Disease course (year)	6.4 (1.0–11.8)	6.2 (1.0–11.8)	6.7 (1.1–11.8)	0.526
Smoking				0.6416802
Mild	203 (72.5%)	98 (70%)	105 (75%)	
Moderate	59 (21.07%)	32 (22.86%)	27 (19.29%)	
Heavy	18 (6.43%)	10 (7.14%)	8 (5.71%)	
Drinking				0.07289003
Mild	234 (83.57%)	110 (78.57%)	124 (88.57%)	
Moderate	36 (12.86%)	24 (17.14%)	12 (8.57%)	
Heavy	10 (3.57%)	6 (4.29%)	4 (2.86%)	
Cardiovascular diseases				0.2203291
Yes	109 (38.93%)	49 (35%)	60 (42.86%)	
No	171 (61.07%)	91 (65%)	80 (57.14%)	
Diabetes mellitus				0.4979995
Yes	74 (26.43%)	40 (28.57%)	34 (24.29%)	
No	206 (73.57%)	100 (71.43%)	106 (75.71%)	
Digestive system diseases				0.3979264
Yes	120 (42.86%)	64 (45.71%)	56 (40%)	
No	160 (57.14%)	76 (54.29%)	84 (60%)	
Urinary system diseases				0.07721267
Yes	95 (33.93%)	40 (28.57%)	55 (39.29%)	
No	185 (66.07%)	100 (71.43%)	85 (60.71%)	
Musculoskeletal diseases				0.6843132
Yes	74 (26.43%)	35 (25%)	39 (27.86%)	
No	206 (73.57%)	105 (75%)	101 (72.14%)	
Mental illness				0.2319005
Yes	40 (14.29%)	24 (17.14%)	16 (11.43%)	
No	240 (85.71%)	116 (82.86%)	124 (88.57%)	
Hoehn-Yahr classification				0.3310189
Stage I	112 (40%)	51 (36.43%)	61 (43.57%)	
Stage II	116 (41.43%)	64 (45.71%)	52 (37.14%)	
Stage III	52 (18.57%)	25 (17.86%)	27 (19.29%)	

**Table 2:** The expression differences of blood markers at baseline between the observation group and the control group.

Variables	All patients (*n* = 280)	Control group (*n* = 140)	Observation group (*n* = 140)	*P*-value
C-reactive protein, CRP (mg/L)	8.6 (4.5–11.6)	8.5 (4.6–11.6)	8.6 (4.5–11.6)	0.928
White blood cell, WBC (10^9/L)	9.8 (6.1–12.9)	9.8 (6.1–12.8)	9.8 (6.3–12.9)	0.779
Neutrophil-to-lymphocyte ratio, NLR	3.8 (1.8–5.5)	3.7 (1.8–5.5)	3.9 (1.9–5.5)	0.144
Platelet-to-lymphocyte ratio, PLR	290.0 (209.3–363.7)	286.6 (209.3–363.7)	292.4 (210.5–360.1)	0.442
Monocyte-to-lymphocyte ratio, MLR	0.5 (0.3–0.6)	0.4 (0.3–0.6)	0.5 (0.3–0.6)	0.24
Triglycerides, TGL (mmol/L)	1.9 (0.9–2.8)	1.8 (0.9–2.8)	2.0 (0.9–2.8)	0.0753
Low-density lipoprotein, LDL (mmol/L)	2.4 (1.1–3.9)	2.4 (1.1–3.8)	2.5 (1.1–3.9)	0.856
High-density lipoprotein cholesterol, HDL (mmol/L)	1.1 (0.7–1.5)	1.1 (0.7–1.5)	1.1 (0.7–1.5)	0.833

### 3.2 Differences in scores of motor symptoms and sleep disorders between the observation group and the control group before and after treatment

Before treatment, there were no significant differences in UPDRS II, UPDRS III (open and closed), MMSE, PDSS, PSQI, and ESS scores between the two groups. After treatment, the observation group had lower UPDRS II score, UPDRS III (open) score, UPDRS III (closed) score, PDSS score, PSQI score, and ESS score than the control group, and higher MMSE score than the control group. These differences were significant, indicating that aDBS showed better efficacy in improving motor symptoms, sleep disorders, and other aspects (Table 3).

**Table 3:** The differences in motor symptom scores and sleep disorder scores between the observation group and the control group before and after treatment.

Variables	All patients (*n* = 280)	Control group (*n* = 140)	Observation group (*n* = 140)	*P*-value
UPDRSII				
Pre-treatment	22 (18–27)	22 (18–27)	22 (18–27)	0.783
Post-treatment	13 (8–17)	13 (8–17)	11 (8–17)	0.00755
UPDRSIII (open period) score				
Pre-treatment	13 (10–16)	13 (10–16)	12 (10–16)	0.0916
Post-treatment	11 (8–14)	11 (8–14)	10 (8–14)	0.0103
UPDRSIII (closed period) score				
Pre-treatment	48 (36–60)	48 (36–60)	48 (36–60)	0.673
Post-treatment	17 (12–21)	17 (12–21)	15 (12–21)	1.30E-06
MMSE				
Pre-treatment	17 (14–20)	18 (14–20)	17 (14–20)	0.312
Post-treatment	22 (17–26)	21 (17–26)	23 (17–26)	0.0023
PDSS				
Pre-treatment	65 (53–76)	64 (53–76)	66 (53–76)	0.104
Post-treatment	41 (31–53)	43 (32–53)	39 (31–53)	0.022
PSQI				
Pre-treatment	11 (8–14)	11 (8–14)	11 (8–14)	0.344
Post-treatment	3 (0–6)	4 (0–6)	3 (0–6)	0.032
ESS				
Pre-treatment	14 (11–17)	15 (11–17)	14 (11–17)	0.159
Post-treatment	7 (2–11)	8 (2–11)	6 (2–11)	0.00243

### 3.3 Univariate logistic regression analysis of factors affecting therapeutic efficacy

The treatment method does not solely determine the treatment effect. Other factors such as age, disease duration, baseline blood marker levels, etc. may also affect the efficacy. Therefore, we divided these factors into two groups: demographic model and blood marker model, and analyzed the impact of factors in both groups on the efficacy. The results of univariate logistic regression indicate that in the demographic model, age BMI、 Disease duration, smoking, alcohol consumption, and Hoehn Yahr staging are negatively correlated factors, while treatment methods are positively correlated factors. In the blood biomarker model, baseline blood indicators such as CRP, NLR, PLR, MLR, TGL, LDL also have a significant negative impact on the improvement of motor symptoms and sleep disorders, while HDL is positively correlated with the improvement of these symptoms. This indicates that higher baseline levels of CRP, NLR, PLR, TGL, LDL, and lower HDL levels are not conducive to aDBS improving motor symptoms and sleep disorders in patients (Table 4).

**Table 4:** Univariate logistic regression analysis of the effects of variables and treatment methods on motor symptoms and sleep disorders in the two groups.

Term	Estimate	StdError	*Z*-value	*p*-value
Demographic model				
Age	−0.029	0.013	−2.192	0.028
Gender	−0.247	0.284	−0.870	0.384
BMI	−0.096	0.043	−2.251	0.024
Disease course	−0.085	0.042	−1.993	0.046
Hoehn-Yahr classification	−0.896	0.194	−4.618	0.000
Smoking	−0.458	0.214	−2.141	0.032
Drinking	−0.698	0.258	−2.703	0.007
Treatment method	0.938	0.283	3.321	0.001
Blood marker model				
CRP	−0.152	0.069	−2.204	0.028
WBC	−0.128	0.067	−1.916	0.055
NLR	−0.423	0.132	−3.206	0.001
PLR	−0.006	0.003	−2.039	0.041
MLR	−3.073	1.560	−1.970	0.049
TGL	−0.552	0.251	−2.197	0.028
LDL	−0.363	0.166	−2.193	0.028
HDL	1.299	0.604	2.152	0.031
Treatment method	0.938	0.283	3.321	0.001

### 3.4 Multivariate logistic regression analysis of factors affecting therapeutic efficacy

**Figure 1:**
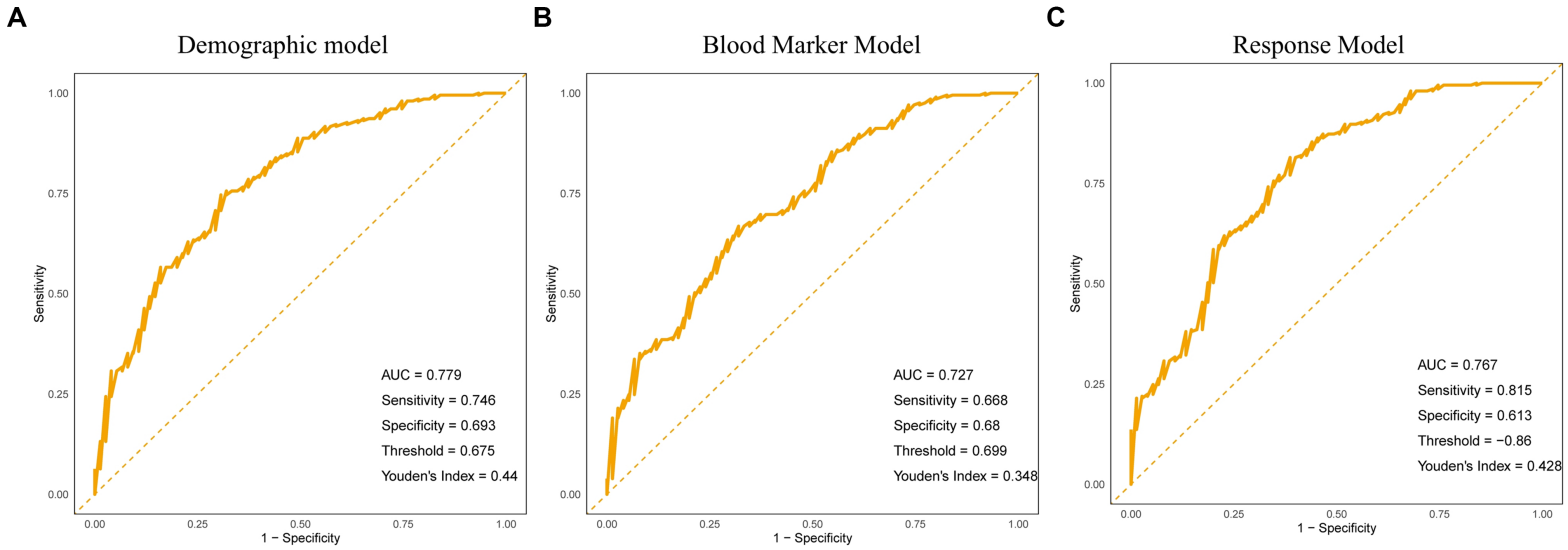
Receiver operating characteristic (ROC) curves of multivariate logistic regression models predicting improvement in motor symptoms and sleep disturbances. **(A)** ROC curve for the demographic model based on multiple demographic variables, including age, gender, body mass index (BMI), and disease duration, among others. **(B)** ROC curve for the blood marker model based on multiple blood markers, including neutrophil-to-lymphocyte ratio (NLR), platelet-to-lymphocyte ratio (PLR), high-density lipoprotein (HDL) levels, among others. **(C)** ROC curve for the combined response model integrating demographic and blood marker factors.

The results showed that age had a significant negative impact on the improvement of motor symptoms and sleep disorders (*p* = 0.014), body mass index (BMI) had a significant negative impact on symptom improvement (*p* = 0.019), disease duration had a significant negative impact on symptom improvement (*p* = 0.013), Hoehn Yahr grading had a significant negative impact on symptom improvement (*p* < 0.001), smoking had a significant negative impact on symptom improvement (*p* = 0.038), and treatment methods had a significant positive impact on symptom improvement (*p* < 0.001). Patients treated with aDBS had a 19% increased likelihood of symptom improvement (OR = 1.190, 95% CI: 1.082–1.310). The neutrophil/lymphocyte ratio (NLR) has a significant negative impact on symptom improvement (*p* = 0.013), and the effect of treatment methods in blood marker models remains significant (*p* = 0.005). Patients treated with aDBS have a 15.5% increased likelihood of symptom improvement (OR = 1.155, 95% CI: 1.045–1.275). In addition, we also compared the performance of two models, and the results showed that the predictive ability of the demographic model was slightly higher than that of the blood biomarker model (AUC 0.779 vs. 0.727), but overall, both models had higher predictive ability (Table 5; Figures 1A,B).

**Table 5:** Multivariate logistic regression analysis of the effects of variables and treatment methods on motor symptoms and sleep disorders in the two groups.

Term	Estimate	SE	Statistic	*p*-value	OR	CI-lower	CI-upper
Demographic model							
Age	−0.006	0.002	−2.485	0.014	0.994	0.990	0.999
Gender	−0.047	0.050	−0.954	0.341	0.954	0.865	1.051
BMI	−0.018	0.008	−2.355	0.019	0.982	0.968	0.997
Disease course	−0.019	0.007	−2.507	0.013	0.981	0.967	0.996
Hoehn Yahr classification	−0.169	0.033	−5.135	0.000	0.845	0.792	0.901
Smoking	−0.084	0.041	−2.082	0.038	0.919	0.849	0.995
Drinking	−0.100	0.051	−1.960	0.051	0.905	0.819	1.000
Treatment method	0.174	0.049	3.579	0.000	1.190	1.082	1.310
Blood marker model							
CRP	−0.014	0.013	−1.080	0.281	0.986	0.962	1.011
WBC	−0.017	0.012	−1.357	0.176	0.983	0.960	1.007
NLR	−0.059	0.024	−2.493	0.013	0.943	0.900	0.987
PLR	−0.001	0.001	−1.728	0.085	0.999	0.998	1.000
MLR	−0.556	0.288	−1.928	0.055	0.574	0.326	1.009
TGL	−0.086	0.046	−1.888	0.060	0.917	0.838	1.003
LDL	−0.038	0.031	−1.240	0.216	0.963	0.907	1.022
HDL	0.168	0.113	1.494	0.136	1.183	0.949	1.476
Treatment method	0.144	0.051	2.835	0.005	1.155	1.045	1.275

### 3.5 Construction of response model

We constructed a response prediction model (referred to as the response model) based on two methods: conventional treatment and aDBS treatment, to predict the improvement of motor symptoms and sleep disorders. We selected significant factors (age, disease duration, Hoehn Yahr grading, baseline NLR level) and treatment methods from the multivariate logistic regression analysis of demographic models and blood marker models, multiplied by the corresponding B values (−0.00589, −0.01875, −0.16895, −0.05902, 0.1590189), and finally added them up to obtain the response model. ROC curve analysis shows that the AUC value of the model is 0.767, indicating a high ability to predict improvement in patients’ motor symptoms and sleep disorders. The threshold is −0.86. When the patient’s score is higher than this, there will be a response to treatment (significant improvement in both motor function and sleep disorders), otherwise there will be no response (Figure 1C).

### 3.6 Generalized linear mixed models are used to analyze the interaction between response model and other factors

For the convenience of analysis, we divided the continuous variables into two categories: those above the median are coded as 0, those below the median are coded as 1 (HDL is the opposite), gender in the categorical variables is coded as 0 for males and 1 for females, mild smoking and alcohol consumption are coded as 2, moderate smoking and alcohol consumption are coded as 1, and severe smoking and alcohol consumption are coded as 0. The results indicate a significant interaction between the response model and BMI grouping, with a regression coefficient of 0.423, a *p*-value of 0.002, and an OR of 1.526 for the interaction term. This suggests that the combination of the response model group and BMI group significantly increases the likelihood of improving motor symptoms and sleep disorders. However, the OR value of the response model group was 6.556, and the OR value of the BMI group was 2.091, indicating that the response model group had a greater impact on improving motor symptoms and sleep disorders than the BMI group, meaning that the effect of the response model was much higher than that of BMI. The OR value of the interaction is smaller than the individual effect of the two, indicating that the addition of BMI increases the probability of symptom improvement, but this increase is not significant enough to amplify the effect of the response model itself, that is, weaken the effect of the response model. There is a significant interaction between the response model group and the PLR group, with an OR value of 4.388, which is higher than their individual effects, indicating that the addition of the PLR group enhances the predictive performance of the response model. There is a significant interaction between the response model group and the HDL group, with an OR value of 1.454, which is lower than the individual effect of the response model group, indicating an antagonistic effect between the two. The addition of the HDL group weakens the predictive effect of the response model (Table 6).

**Table 6:** Generalized linear mixed model analysis of the interaction between the response model and other factors.

Term	Estimate	Std error	Statistic	*p*-value	OR	CI-lower	CI-upper
Reference	0.243	0.211	1.150	0.250	1.275	0.843	1.929
Response model group	1.759	0.398	4.415	0.000	5.804	2.659	12.670
Gender	0.301	0.364	0.826	0.409	1.351	0.662	2.757
Response model group * gender	−0.356	0.640	−0.557	0.578	0.700	0.200	2.454
Reference	0.000	0.236	0.000	1.000	1.000	0.630	1.587
Response model group	1.880	0.429	4.388	0.000	6.556	2.830	15.183
BMI group	0.738	0.350	2.105	0.035	2.091	1.052	4.155
Response model group * BMI group	0.423	0.135	3.128	0.002	1.526	1.171	1.989
Reference	−0.988	0.590	−1.674	0.094	0.372	0.117	1.184
Response Model group	2.499	0.964	2.591	0.010	12.166	1.838	80.524
Smoking	0.795	0.347	2.287	0.022	2.213	1.120	4.373
Response model group * smoking	−0.500	0.516	−0.969	0.333	0.607	0.221	1.667
Reference	−0.935	0.618	−1.513	0.130	0.393	0.117	1.318
Response model group	3.198	1.573	2.034	0.042	24.478	1.123	533.722
Drinking	0.740	0.352	2.102	0.036	2.097	1.051	4.183
Response model group * drinking	−0.891	0.805	−1.107	0.268	0.410	0.085	1.987
Reference	0.000	0.232	0.000	1.000	1.000	0.634	1.577
Response model group	1.846	0.429	4.305	0.000	6.333	2.733	14.675
CRP group	0.762	0.352	2.163	0.031	2.143	1.074	4.275
Response model group * CRP group	−0.498	0.627	−0.794	0.427	0.608	0.178	2.077
Reference	0.167	0.237	0.706	0.480	1.182	0.743	1.879
Response model group	1.713	0.429	3.994	0.000	5.547	2.393	12.859
WBC group	0.375	0.345	1.087	0.277	1.455	0.740	2.863
Response model group * WBC group	−0.176	0.623	−0.283	0.777	0.838	0.247	2.842
Reference	0.279	0.250	1.113	0.266	1.321	0.809	2.159
Response model group	1.193	0.388	3.075	0.002	3.297	1.541	7.054
PLR group	0.127	0.344	0.369	0.712	1.135	0.578	2.228
Response model group * PLR group	1.479	0.720	2.053	0.040	4.388	1.069	18.009
Reference	0.158	0.230	0.687	0.492	1.171	0.746	1.839
Response model group	1.652	0.428	3.863	0.000	5.217	2.256	12.061
MLR group	0.420	0.348	1.208	0.227	1.522	0.770	3.008
Response model group * MLR group	−0.090	0.624	−0.144	0.886	0.914	0.269	3.108
Reference	0.029	0.241	0.120	0.904	1.029	0.642	1.650
Response model group	1.779	0.418	4.260	0.000	5.926	2.614	13.435
TGL group	0.643	0.348	1.849	0.064	1.902	0.962	3.761
Response model group * TGL group	−0.270	0.630	−0.429	0.668	0.763	0.222	2.621
Reference	−0.059	0.243	−0.243	0.808	0.943	0.586	1.517
Response model group	1.571	0.391	4.021	0.000	4.814	2.238	10.355
LDL Group	0.815	0.350	2.327	0.020	2.260	1.137	4.490
Response model group * LDL group	0.445	0.694	0.641	0.522	1.560	0.400	6.086
Reference	0.158	0.230	0.688	0.492	1.171	0.746	1.839
Response model group	1.939	0.462	4.197	0.000	6.951	2.811	17.192
HDL group	0.420	0.348	1.208	0.227	1.522	0.770	3.008
Response model group * HDL group	0.374	0.117	3.187	0.001	1.454	1.155	1.830

### 3.7 Analysis of quality of life and patient satisfaction between the observation group and the control group

**Figure 2:**
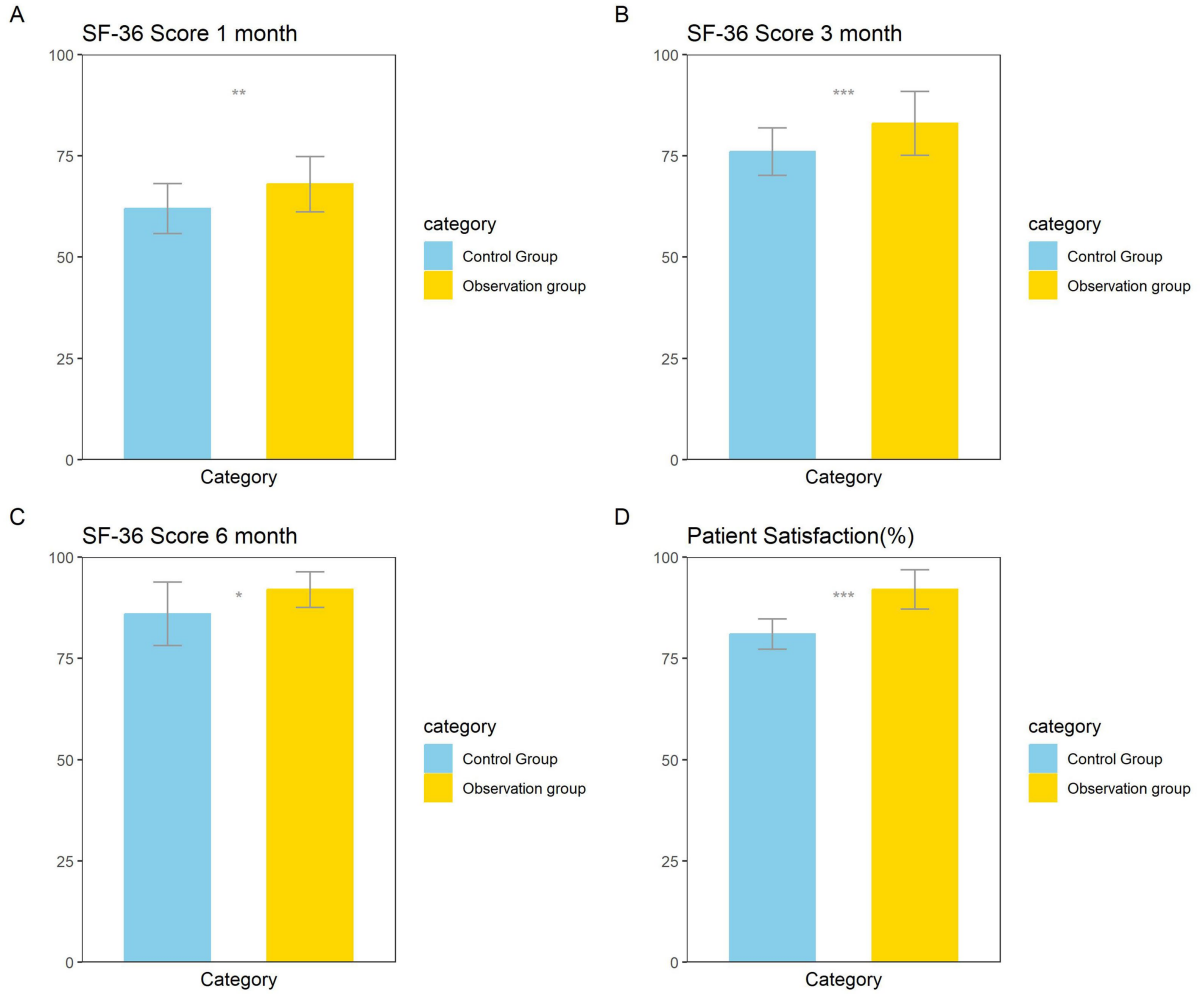
Differences in SF-36 scores between the observation group and the control group at **(A)** 1 month, **(B)** 3 months, and **(C)** 6 months post-treatment. **(D)** Differences in patient satisfaction between the observation group and the control group. *p < 0.05, **p < 0.01, ***p < 0.001.

The results showed that the quality of life of the observation group was significantly higher than that of the control group at 1, 3, and 6 months after treatment. Patient satisfaction was significantly higher than that of the control group (Figures 2A–D).

## 4 Discussion

Our research found that adaptive deep brain stimulation (aDBS) improves motor function and sleep disorders in patients with Parkinson’s disease, likely because aDBS delivers more precise electrical stimulation to specific brain regions, such as the subthalamic nucleus and internal capsule, thereby restoring neural circuit function (14). In Parkinson’s disease patients, electrical stimulation of the medial thalamus and subthalamic nuclei effectively inhibits excessive basal ganglia activity, reducing motor symptoms. Simultaneously, aDBS also influences brain areas involved in sleep regulation by stabilizing neural circuit discharge patterns, which helps alleviate sleep problems such as insomnia and daytime sleepiness. A key advantage of aDBS lies in its high degree of individualization and adaptability. By precisely adjusting stimulation frequency, amplitude, and pulse width, aDBS provides personalized treatment tailored to the patient’s condition and symptom profile, thereby optimizing clinical outcomes (15).

The response model constructed in this study holds significant clinical value. It can predict whether a patient will respond to adaptive deep brain stimulation (aDBS) treatment based on the treatment method and several baseline factors. For instance, if a patient undergoes aDBS, variables such as age, disease duration, Hoehn-Yahr stage, and baseline neutrophil-to-lymphocyte ratio (NLR) can be used to assess the likelihood of effective improvement in motor function and sleep disorders. A final score above −0.83 indicates that the patient is a suitable candidate for aDBS therapy. This model therefore reduces the uncertainty and randomness in treatment selection, enhancing treatment precision. By utilizing this model, clinicians can better predict treatment outcomes, optimize therapeutic decisions, and ultimately improve patients’ quality of life and satisfaction with care.

A major strength of this study is the use of generalized linear mixed models (GLMM) for interaction analysis. In medical research, data often exhibit a hierarchical structure—for example, measurements taken at multiple time points from the same patient and variability between different patients. GLMMs effectively accommodate such hierarchical data by accounting for both fixed effects and random effects, the latter representing individual differences among patients. This is particularly important because patient responses to treatment can vary widely (16). These variations are influenced not only by known baseline factors such as age and gender but also by numerous unknown factors. Incorporating random effects allows for quantification of these individual differences, providing a more comprehensive analysis. This approach distinguishes our study from previous interaction analyses.

Our study suggests that patients with lower baseline platelet-to-lymphocyte ratio (PLR) exhibit a stronger response to adaptive deep brain stimulation (aDBS) treatment. This may be because a lower PLR reflects reduced inflammation and a healthier immune status (17, 18), which enhances aDBS’s regulatory effects on the nervous system. Patients with low inflammation can better adapt their neural circuits to aDBS stimulation, leading to significant improvements in motor symptoms and sleep quality. Conversely, patients with lower body mass index (BMI) show a weaker response to aDBS, possibly due to insufficient nutritional and energy reserves, as well as reduced fat and muscle mass, resulting in poorer adaptability to the treatment and diminished effectiveness. Additionally, patients with higher baseline high-density lipoprotein (HDL) levels also demonstrate a weaker response to aDBS. Although HDL is generally regarded as “good” cholesterol that facilitates cholesterol clearance and reduces inflammation, it also has neuroprotective properties (19, 20). However, in Parkinson’s disease patients, excessively high HDL levels may interfere with certain neural repair mechanisms or the neural adaptation processes triggered by electrical stimulation, thereby weakening the treatment response.

Similarly, this study also has certain limitations. Firstly, as a retrospective study, the data comes from existing clinical cases or treatment records, which may lead to data selection bias. Secondly, the mechanism of aDBS treatment response in patients with lower baseline PLR levels, lower BMI, and higher baseline HDL levels needs to be further validated through experimental studies.

## 5 Conclusion

This study found that aDBS significantly improved the motor function and sleep disorders of Parkinson’s disease patients, and constructed a response model that can effectively predict the treatment effect of Parkinson’s disease patients. It was also found that the response model was correlated with baseline PLR, BMI. There is a significant interaction between baseline HDL. This study can help clinical doctors more accurately evaluate the treatment prognosis of patients before treatment, thereby achieving personalized treatment and improving treatment effectiveness. And this study also provides basic data for further exploring the mechanism of aDBS treatment and its relationship with biomarkers, which has important clinical application value.

## Data Availability

The original contributions presented in the study are included in the article/supplementary material, further inquiries can be directed to the corresponding author.
